# A Comprehensive Study of N-Butyl-1H-Benzimidazole

**DOI:** 10.3390/molecules27227864

**Published:** 2022-11-14

**Authors:** Aleksandr S. Kazachenko, Emine Tanış, Feride Akman, Mouna Medimagh, Noureddine Issaoui, Omar Al-Dossary, Leda G. Bousiakou, Anna S. Kazachenko, Dmitry Zimonin, Andrey M. Skripnikov

**Affiliations:** 1 School of Non-Ferrous Metals and Material Science, Siberian Federal University, Pr. Svobodny 79, 660041 Krasnoyarsk, Russia; kaalla@list.ru (A.S.K.);; 2 Institute of Chemistry and Chemical Technology, Krasnoyarsk Scientific Center, Siberian Branch, Russian Academy of Sciences, Akademgorodok, 50, Bld. 24, 660036 Krasnoyarsk, Russia; 3 Department of Biological Chemistry with Courses in Medical, Pharmaceutical and Toxicological Chemistry, Krasnoyarsk State Medical University of the Ministry of Healthcare of the Russian Federation, St. Partizan Zheleznyak, Bld. 1, 660022 Krasnoyarsk, Russia; 4 Department of Electrical Electronics Engineering, Faculty of Engineering and Architecture, Kırşehir Ahi Evran University, Kırşehir 40100, Turkey; 5 Vocational School of Food, Agriculture and Livestock, University of Bingöl, Bingöl 12000, Turkey; 6 Laboratory of Quantum and Statistical Physics (LR18ES18), Faculty of Sciences, University of Monastir, Monastir 5000, Tunisia; 7 Department of Physics and Astronomy, College of Science, King Saud University, P.O. Box 2455, Riyadh 11451, Saudi Arabia; 8 IMD Laboratories Co., R&D Section, Lefkippos Technology Park, NCSR Demokritos, P.O. Box 60037, 15130 Athens, Greece

**Keywords:** N-butyl-1H-benzimidazole, benzimidazole, DFT, AIM, RDG, ELF

## Abstract

Imidazole derivatives have found wide application in organic and medicinal chemistry. In particular, benzimidazoles have proven biological activity as antiviral, antimicrobial, and antitumor agents. In this work, we experimentally and theoretically investigated N-Butyl-1H-benzimidazole. It has been shown that the presence of a butyl substituent in the N position does not significantly affect the conjugation and structural organization of benzimidazole. The optimized molecular parameters were performed by the DFT/B3LYP method with 6-311++G(d,p) basis set. This level of theory shows excellent concurrence with the experimental data. The non-covalent interactions that existed within our compound N-Butyl-1H-benzimidazole were also analyzed by the AIM, RDG, ELF, and LOL topological methods. The color shades of the ELF and LOL maps confirm the presence of bonding and non-bonding electrons in N-Butyl-1H-benzimidazole. From DFT calculations, various methods such as molecular electrostatic potential (MEP), Fukui functions, Mulliken atomic charges, and frontier molecular orbital (HOMO-LUMO) were characterized. Furthermore, UV-Vis absorption and natural bond orbital (NBO) analysis were calculated. It is shown that the experimental and theoretical spectra of N-Butyl-1H-benzimidazole have a peak at 248 nm; in addition, the experimental spectrum has a peak near 295 nm. The NBO method shows that the delocalization of the aσ-electron from σ (C1–C2) is distributed into antibonding σ* (C1–C6), σ* (C1–N26), and σ* (C6–H11), which leads to stabilization energies of 4.63, 0.86, and 2.42 KJ/mol, respectively. Spectroscopic investigations of N-Butyl-1H-benzimidazole were carried out experimentally and theoretically to find FTIR vibrational spectra.

## 1. Introduction

Heterocyclic compounds are important substances. They are widely distributed in nature, and are important in the metabolism of all living cells and in the chemistry of natural compounds. Aromatic nitrogen-containing heterocyclic compounds are the most diverse and well-studied. Nitrogen-based heterocyclic compounds play an important role for humanity. In particular, benzimidazole is of great importance, not only biologically but also industrially among all nitrogen-based heterocyclic compounds [1,2]. Benzimidazole is a nitrogen-containing aromatic heterocyclic compound whose structure includes conjugated rings of benzene and imidazole.

Benzimidazoles are heterocyclic compounds that have attracted great interest in the last few years due to their proven biological activity such as antiviral [3], antitumor [4], antifungal [5], antimicrobial [6], and other substances [7]. Also, benzimidazoles are widely used in industrial processes as corrosion inhibitors for surfaces of metals and alloys [8].

Benzimidazole derivatives have different types of pharmacokinetic and pharmacodynamic properties. The benzimidazole core is one of the bioactive heterocyclic compounds exhibiting a number of biological activities [9].

It was shown [1,2] that the functional group present in the benzimidazole molecule plays an important role in the physicochemical properties of the molecule. To determine the best molecule for a therapeutic target (as well as the required physicochemical characteristics), it is necessary to understand the relative contribution of each functional group. The benzimidazole molecule has proven to be important in biochemistry due to its biological activity and simple compound. Recently, new medically important benzimidazole derivatives have been synthesized.

The physical and chemical properties of imidazoles and benzimidazoles, which are potential starting materials for a large number of important chemicals, are currently being actively studied. Considering that even the most fundamental physical properties of alkylimidazoles are actively investigated to this day, there is still insufficient data for many benzimidazole derivatives [10,11]. The biological activity of benzimidazole derivatives is influenced by both the type of substituent and the position at which it is attached to the benzimidazole ring [12].

N-Butyl-1H-benzimidazole (1-Butylbenzimidazole) is an important imidazolium derivative. It and its analogs have been investigated as an electrolyte with improved charge transfer capabilities for dye-sensitized solar cells [13] and for the synthesis of N-(fluoroalkyl)imidazolones [14].

In addition, N-Butyl-1H-benzimidazole can be a platform molecule for obtaining brønsted acidic ionic liquid, which can be used in esterifications of aliphatic acids [11] and others [15,16].

Since the physical and chemical properties of chemicals are decisive in their practical application, an urgent task is to study them by various methods, both experimental and theoretical. The aim of this work was to study N-Butyl-1H-benzimidazole using FTIR spectroscopy, Mulliken atomic charges, UV-Vis, DFT, RDG, QTAIM, ELF, LOL, NBO, HOMO-LUMO, Fukui functions, and MEP.

## 2. Result and Discussion

### 2.1. Structural Analysis of N-Butyl-1H-Benzimidazole

In this work, we used the DFT and QTAIM method with B3LYP/6-311++G(d,p) for calculation of the benzimidazole derivative.

It is known [17] that the physicochemical properties of imidazole derivatives of this type are affected by the following factors: the charge distribution on ions, H bond, ion symmetry, and van der Waals interaction. The size of the alkyl chain also plays an important role in determining the characteristics of ionic liquids. For example, it is known [18] that with an increase in the length of the alkyl chain, the melting point of ionic liquids gradually decreases.

**Figure 1:**
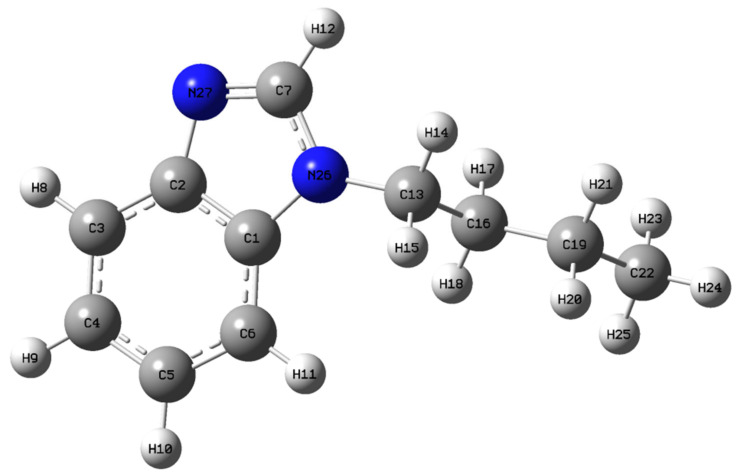
Optimized molecular structure of N-Butyl-1H-benzimidazole.

In our case, imidazole has a butyl and benzyl substituent and its optimized structure is shown in Figure 1, and the optimized parameters are shown in Table 1.

Comparison of the optimized structural parameters of N-Butyl-1H-benzimidazole showed that the C1-N26 and C2-N27 bonds have similar values (1.386 and 1.387 Å, respectively) for theoretical calculations. The experimental values for C1-N26 and C2-N27 are 1.391 and 1.367 Å, respectively. Benzimidazole was calculated in [19] using the DFT method with B3LYP functional and the 6-311G+(2d,p) basis set. It was shown that for bonds analogous to C1-N26 and C2-N27 (N-Butyl-1H-benzimidazole), the bond lengths are 1.389 and 1.385 Å, respectively.

The C7-N26 and C7-N27 bond lengths are 1.377 and 1.306 Å, respectively. For benzimidazole, the C7-N26 and C7-N27 bond lengths are 1.377 and 1.304 Å, respectively [19]. Thus, the lengths of bonds C1-N26, C2-N27, C7-N26, and C7-N27 in benzimidazole and N-Butyl-1H-benzimidazole are similar, which allows us to judge that the butyl substituent has almost no effect on this type of bond. For the butyl group, the bond lengths C13-C16, C16-C19, and C19-C22 are 1.534, 1.533, and 1.531 Å, respectively.

According to Table 1, in general, for the experimental values of the bond lengths, lower values are observed in comparison with the theoretical ones.

The value of the angles with the C-N-C and N-C-N bonds obtained using theoretical calculations differs from the experimental data (Table 1), which may be due to a systematic calculation error [20,21,22]. Thus, for N26-C7-N27, C2-N27-C7, and C1-N26-C7, the theoretical angles are 114.3, 104.6, and 105.9°, respectively, and for the experimentally determined angles N26-C7-N27, C2-N27-C7, and C1-N26-C7, the values are 121.6, 105.4, and 109.5°, respectively. The obtained theoretical values of the angles are consistent with the literature data on benzimidazole [19] for which the angles N26-C7-N27, C2-N27-C7, and C1-N26-C7 are 113.4, 104.9, and 106.8°, respectively.

Thus, the introduction of an alkyl group into the N position in benzimidazole has almost no effect on the C-N bond lengths and C-N-C and N-C-N angles, which is in good agreement with the results presented in [19].

### 2.2. Topological Analysis

Topological analysis helps to describe and predict the molecular structure of molecules, and also provides a model for explaining how aetheric wave functions of atoms should fit together [23].

The theory of atoms in molecules (AIM) [24,25] provides a rigorous solution to the problem of dividing each molecular property into contributions from atoms or functional groups. Using this theory, one can obtain data on the topological characteristics of the studied substances [24,26,27]. In addition, this technique is an appropriate method to examine the intra- and intermolecular interactions in terms of the electron density of a molecular system [28].

In the AIM theory, the character of the bond is described by the electron density ρ(r), the kinetic energy density G(r), the electron density Laplacian ∇^2^ρ(r), the potential energy density V(r), the interaction energy Einteractions, and the ratio │V│/G. However, the values |V(r)|/G(r) make it possible to judge the nature of interactions: for covalent bonds, this ratio is greater than 2; for mixed interactions it is between 1 and 2; and for ionic bonds, van der Waals interactions have a value less than 1 [29].

**Figure 2:**
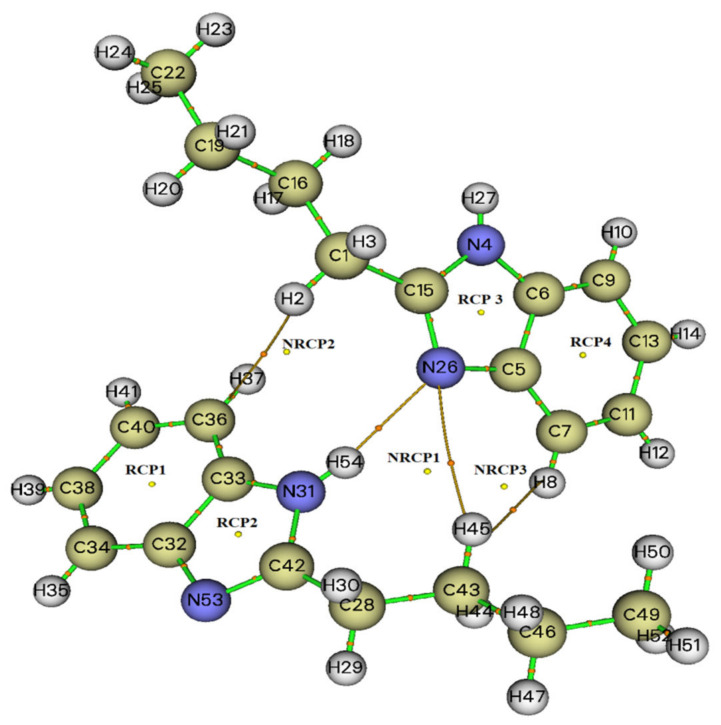
Molecular graph of the dimer.

To determine the electronic structure and obtain the topological characteristics of N-Butyl-1H-benzimidazole, the method of quantum theory of atoms in a molecule (QTAIM) was used. The molecular graph of the dimer is shown in Figure 2.

In addition, the topological parameters in the BCP of the cation around the atoms were calculated; the results are presented in Table 2. The ratios |V(r)|/G < 1, H(r) > 0, and ∇^2^ρ(r) > 0 for all types (except RCP2) indicate closed-shell interactions typical of ionic bonds and van der Waals interactions.

According to Table 2, the largest value of ∇^2^ρ(r) is observed for NRCP1 (0.3993 a.u.), and the smallest for RCP2 (−0.0176 a.u.). It should be noted that the maximum values of ρ(r) and G(r) are observed for RCP2 and are 0.3888 and 0.3888 a.u., respectively, and also for this type of interaction, the minimum values of V(r) (−0.6782 a.u.), H(r) (−0.5589 a.u.), and Einteractions (−890.05 kJ/mol) are observed.

Similar and comparable values are shown of C-H…H: ∇^2^ρ(r) (0.0063–0.0069 a.u), ρ(r) (0.0017-0.0019 a.u), G(r) (−0.0008–0.0009 a.u), H(r) (0.0004 a.u), and E_interactions_ −1.05 kJ/mol. It should be noted that the interaction between hydrogen and nitrogen in C-H…N and N-H…N differ significantly in all of the main parameters.

The topological analysis of the electron localization function ELF gives a division of the molecular space into basins of attractors that have a clear chemical significance [30]. The hierarchy of these basins is given by the bifurcation of localization domains. In the case of π-donor substituents (OH, NH_2_, F, CH_3_, etc.), the aromatic domain opens first near the substituted carbon and then near the metacarbon [31]. The orienting effects of electrophilic substitutions correlate with these bifurcations.

Analysis of the ELF function allows the division of the molecular space not into an atomic pool, as in the Bader theory, or into areas of charge concentration, but into electron localization pools, inside which the excess of kinetic energy due to Pauli repulsion is minimal. The spatial position of these attractors makes it possible to distinguish between core and valence basins [31,32]. The pools of the center are located around the nuclei (except for the hydrogen atom). Valence basins are classified according to their relationship to the main basins. The topological analysis of the electron localization function is a suitable mathematical model for characterizing chemical bonds [33].

**Figure 3:**
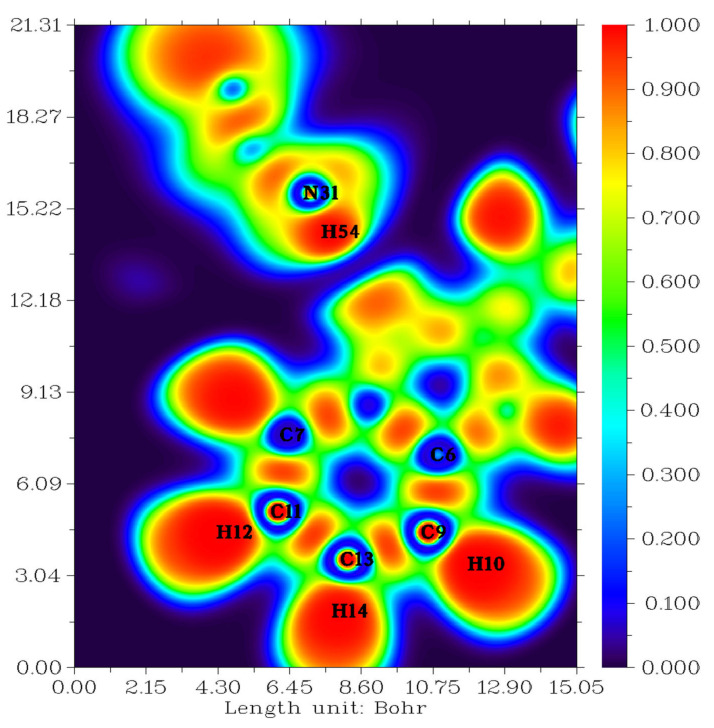
Electron localization function (ELF) map of the title compound.

The ELF isosurface representation for N-Butyl-1H-benzimidazole was determined using the Multiwfn program [34]. Figure 3 shows shaded surface maps with an electron localization function (ELF) projection effect. As you can see, several colors are represented on this surface. The red and orange colors represent strong electronic localization. The blue-colored circle represents the depletion region between the inner shell and the valence shell. The hydrogen and carbon regions have the minimum values of the localized orbital locator.

Topological analysis of the electron localization function (ELF) and localized orbital locator (LOL) are tools used to perform the analysis of covalent bonds since they reveal regions of molecular space where the probability of detecting a pair of electrons is high [35].

**Figure 4:**
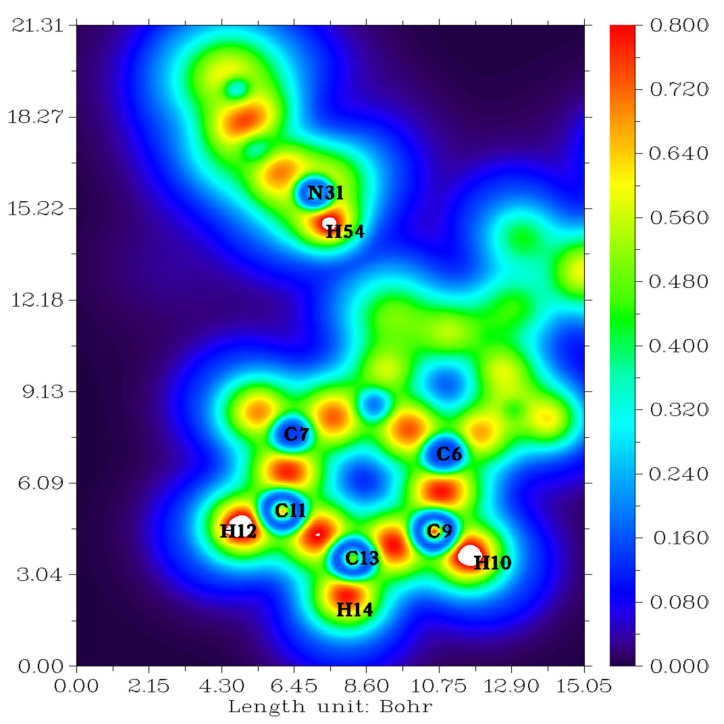
Localized orbital locator (LOL) for our compound.

ELF and LOL have a homogeneous chemical composition as they depend on the kinetic energy density. ELF explains the density of electron pairs, while LOL explains the maximum overlap of localized orbitals due to the orbital gradient [36]. The meanings of ELF and LOL complement each other. The color shades of the ELF and LOL maps shown in Figure 3 and Figure 4 confirm the presence of bonding and non-bonding electrons, where the red color around the hydrogen atoms (H10, H12, H14, H54) with a maximum value indicates the presence of bonding and non-bonding electrons. High ELF or LOL values shown in red around the hydrogen atoms indicate high electron localization due to the ubiquity of a covalent bond, lone electron pair, or nuclear shell in this region, also influenced by the presence of a benzene ring. The C-N chemical bond is described by mislocalization domains (orange) with lower electron localization values [37]. The central region of the hydrogen atom in LOL has a white color since the electron density exceeds the upper limit of the color scale [35].

To investigate the weak interactions within the molecular system, the reduced density gradient (RDG) method was applied. This approach is a topological tool that reveals non-covalent interactions such as van der Waals, hydrogen bonds, and steric collisions. The area of these interactions and their graphical visualization is provided by the XRD analysis based on the electron density and its derivatives [38].

The reduced density gradient (RDG) is a basic non-dimensional quantity consisting of the density and first derivative and denoted as follows [39]:

(1) RDGr=123π213∇ρrρr43  

**Figure 5:**
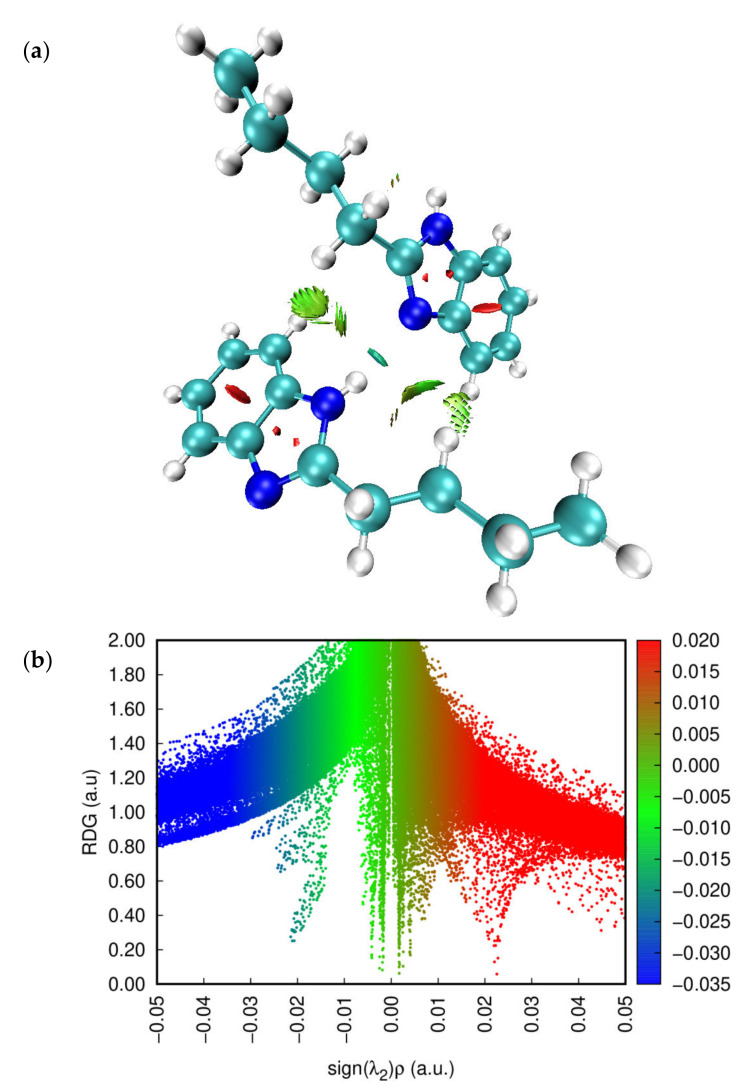
(**a**) VMD graph and (**b**) RDG scatter map of the title compound.

The graphical representation of ρ(r) as a function of the sign of (λ2) ρ, where the sign of (λ2) ρ is the second eigenvalue of the electron density, provides convenient information about the strength and nature of interactions [40]. The interactions of repulsion, attraction, and van der Waals correspond to sgn(λ2) ρ > 0, sgn(λ2) ρ < 0, and sgn(λ2) ρ ≈ 0, respectively [41]. The 2D scatterplot and 3D RDG isosurface densities for N-Butyl-1H-benzimidazole are shown in Figure 5.

As can be seen in Figure 5, the blue color shows the hydrogen bond, while the green color corresponds to the van der Waals interaction, and the red color to the steric cyclic effect. The results of the RDG analysis show that there are attractive, van der Waals, and repulsive interactions in N-Butyl-1H-benzimidazole. In addition, NCI numbers are used to visualize the corresponding strength of H bonds, and plots for the systems studied are shown in Figure 5. As you can see, disc-shaped blocks, which indicate non-covalent interactions and the strongest H bonds, are indicated in blue-green.

The molecular electrostatic potential is very informative in relation to the distribution of the nuclear and electronic charge of molecules; in addition, it is a tool for interpreting and predicting chemical activity, as well as the interaction of hydrogen bonds [42].

### 2.3. MEP Analysis

Molecular electrostatic potential maps, also known as electrostatic potential energy or molecular surface electric potential maps, illustrate the charge distribution of molecules in three dimensions [43], which is used to determine how molecules interact with each other [40]. Molecular electrostatic potential maps help visualize the charge distribution of molecules and charge-related properties of molecules, as well as the size and shape of molecules. Electrostatic potential analysis is related to electron density and is very useful for assessing the position of electrophilic and nucleophilic attacks, as well as the interaction of hydrogen bonds [44,45,46].

**Figure 6:**
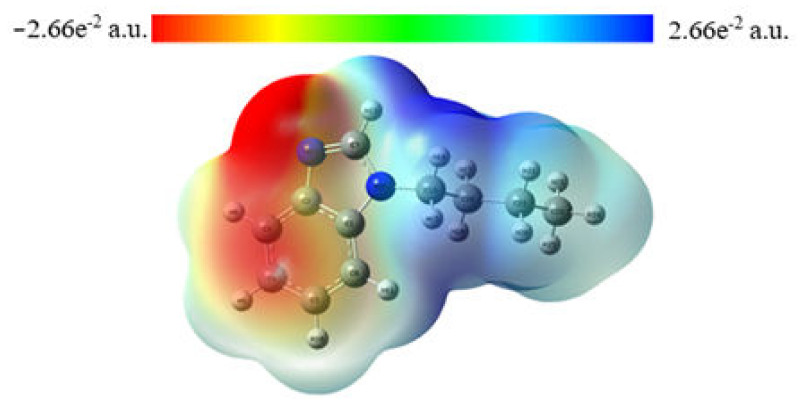
Molecular electrostatic potential map.

Different colors in the MEP (Figure 6) indicate different electrostatic potential values. The descending order potential is expressed as follows: blue > green > yellow > orange > red. Negative values in Figure 6 are shown in red and are associated with the area of electrophilic attack. The area of nucleophilic attack (positive area) is shown in blue and is mainly related to the N26 nitrogen atoms.

### 2.4. Frontier Molecular Orbital (FMO) Analysis

In computational chemistry, the energy gap of HOMO-LUMO and the electronic proprieties of the FMOs are very imperative descriptors [47]. This method is widely used to explain both the electronic and optical properties of compounds. The main participants in molecular interactions are the highest occupied molecular orbital (HOMO) and the lowest unoccupied molecular orbital (LUMO). These values are used to determine the kinetic stability and chemical reactivity of molecules. The LUMO energy level represents the electron-withdrawing abilities, while the HOMO shows the electron-donating ability [48,49,50]. The exact energies of HOMO and LUMO are the ionization potential and electron affinity, respectively [51]. A molecule with a small energy gap means it is highly polarized and is mainly associated with high chemical reactivity and low kinetic stability [52,53].

The electronic properties of various benzimidazoles and their derivatives are being actively studied [54,55]. For example, the authors studied in detail (wavelength, Osc strength (f), orbital composition to the electronic transitions) the ruthenium complex of tetradentate N,N′-bis(benzimidazole-2yl-ethyl)-ethylenediamine [56].

**Figure 7:**
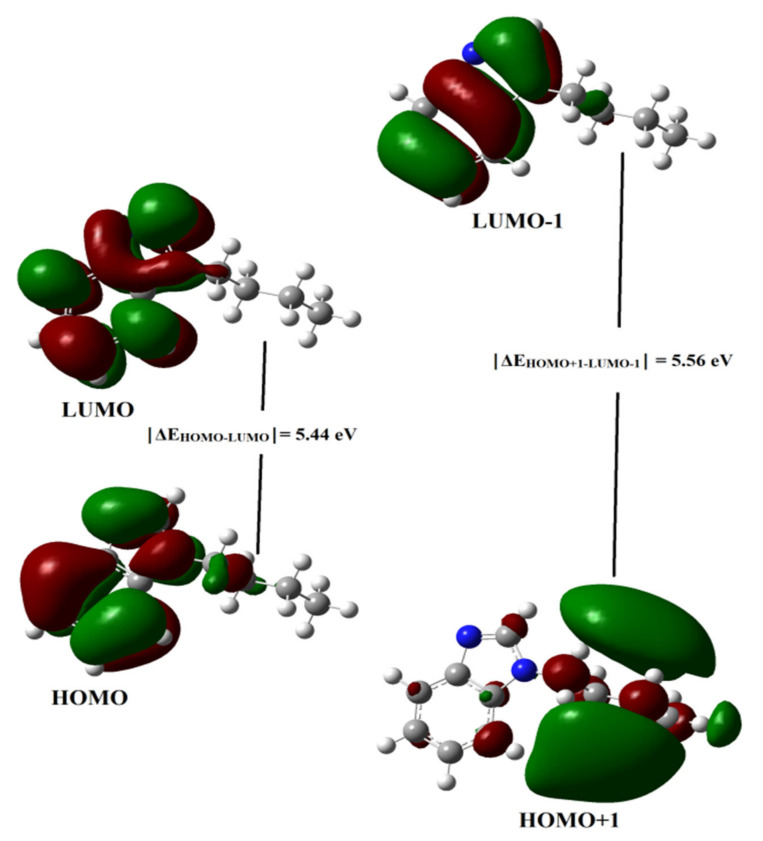
Frontier molecular orbital (HOMO-LUMO) image of the title compound.

Graphs of frontier molecular orbitals (HOMO and LUMO) of N-Butyl-1H-benzimidazole were calculated by the DFT/B3LYP/6-31++G(d, p) method used in the optimization of molecules and are shown in Figure 7.

As shown in Figure 7, HOMO and LUMO have nodes and are arranged symmetrically. Red is the positive phase, green is the negative phase. Using the HOMO and LUMO energies, the following were calculated: electron affinity (EA), electronegativity (χ), energy gap (Egap), chemical potential (μ), hardness (η), ionization potential (IP), softness (ς), and electrophilicity index (ɷ) for N-Butyl-1H-benzimidazole using procedures in [57]. The data are presented in Table 3.

According to Table 3, the chemical potential of N-Butyl-1H-benzimidazole has a negative value, i.e., the molecule is stable. In other words, it does not spontaneously disintegrate into elements. Hardness indicates the resistance of chemical systems to deformation of the electron cloud during chemical treatment [58]. Hard systems with a large HOMO-LUMO energy gap are much less polarizable and relatively small, while soft systems with a small HOMO-LUMO energy gap are strongly polarizable and large [52].

### 2.5. Fukui Functions

Fukui functions are defined as a measure of the sensitivity of a particular region of a N-electron system to an external chemical potential [52,59,60,61]. Therefore, Fukui functions are used when determining atomic centers with high regional electrophilic reactivity, local nucleophilic reactivity, and local radical reactivity in a molecule. The Fukui functions (f^+^ (r), f^−^ (r), f^0^ (r)) defined by Kolandaivel et al. [62] are calculated using the following equations:

f^+^ (r) = q_(N+1)_ (r) − q_(N)_ (r) for nucleophilic attack (2)

f^−^ (r) = q_(N)_ (r) − q_(N−1)_ (r) for electrophilic attack (3)

f^0^ (r) = 1/2 [q_(N+1)_ (r) − q_(N−1)_ (r)] for radical attack (4)

In the above equations, q (r) represents the atomic charge obtained from the electrostatically-derived charge, and the Mulliken population analysis for neutral (N), anionic (N−1), and cationic (N+1) chemical structures at the nth atomic site. +, −, and 0 signs represent nucleophilic, electrophilic, and radical attack, respectively. The data for the Fukui functions are listed in Table S1. The high value of the Fukui function of an atom indicates that the molecular reactivity is also high [63,64]. Compared to these three attacks, the molecule is more nucleophilic. The dual identifier Δf(r) in the last row of Table S1 is the difference between their signs and nucleophilic and electrophilic attacks in a particular region and is calculated with the following equation:

Δf(r) = f^+^ (r) − f^−^ (r) (5)

If Δf(r) > 0, the site can be considered a nucleophilic attack, and when Δf(r)<0, the site can be considered an electrophilic attack [65]. As shown in Table S1, the nucleophilic case is in the order C22>C13>H17>C2>C5>C4>H15>C6>C16>C19>N27>H9>H8. The electrophilic case is in the order H24>H21>H20>H23>H11>H12>H14>H25>C1>H18>C7>C3>H10.

### 2.6. UV-Vis Analysis and NBO Analysis

UV spectrophotometry helps to identify organic compounds and observe some changes during their chemical modification [66,67].

It is known [19] that the UV-visible spectra of benzimidazole and its derivatives are characterized by four sets of bands. The absorption band of the lowest energy electronic transition (π→π*) at 278 nm in benzimidazole was modified by the addition of substituents causing a shift corresponding to the lower energy LUMO stabilized by N-substitution.

This absorption shift is likely due to an increase in chromophore conjugation. Extensive delocalization of π-electrons in alkyl-substituted benzimidazole can be associated with hyperconjugation, in which σ-electrons of the alkyl bond participate in resonance with the benzimidazole ring [19].

**Figure 8:**
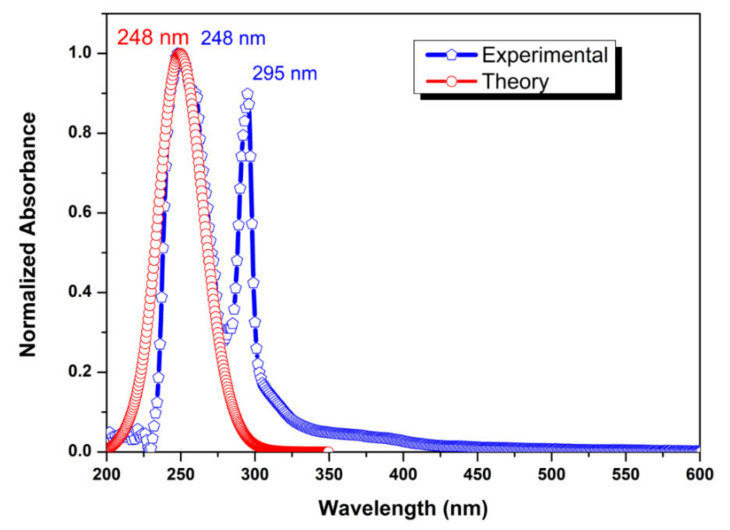
UV-Vis spectra of the title compound.

The UV-Vis spectrum of N-Butyl-1H-benzimidazole is shown in Figure 8. It is shown that the experimental and theoretical spectra of N-Butyl-1H-benzimidazole have a peak at 248 nm, in addition, the experimental spectrum has a peak at 295 nm. The presence of an additional peak at 295 may be due to both the influence of the solvent and the transition (π→π*) in the benzimidazole fragment [68].

NBO analysis is an efficient method that effectively describes bond-to-bond interactions, charge transfer or conjugation in molecular systems, as well as various second-order interactions between occupied and vacant orbitals, and provides detailed information on intramolecular and intermolecular hydrogen bonds [69].

Natural bond orbit analysis (NBO) is one of the many options available to “translate” the calculated solutions of the Schrödinger wave equation into the familiar language of chemical bond concepts. This method has important features such as broad agreement (including with respect to experimental data), good predictive ability, including numerical model predictions, and others [70]. The NBO analysis is performed by calculating the stabilization energies (E^(2)^) using the following equation:

(6) $$E^{\left(2\right)}=\Delta E_{ij}=q_{i}\frac{F{\left(i,j\right)}^{2}}{\varepsilon_{j}-\varepsilon_{i}}$$

where F(i, j), ε_j_, and ε_i_, q_i_ are the diagonal NBO Fock matrix element, the diagonal elements, and the donor orbital occupancy, respectively.

To determine the nature of the interaction that exists within N-Butyl-1H-benzimidazole and the charge distribution on the ion pairs, NBO analysis was performed on the optimized structure (Table S2).

The importance of hyperconjugative interaction and the transfer of electron density from lone pair electrons to the antibonding orbital was analyzed [71].

Intramolecular interactions with charge transfer for the most significant stabilization energies E ^(2)^, obtained from NBO calculations, are presented in Table S2. The larger the value of E ^(2)^, the greater the degree of conjugation of the entire system [72,73]. The stabilization of the structure of N-Butyl-1H-benzimidazole is evidenced by the strong intramolecular interaction of σ- and π-electrons of donor C–N, C–C bonds with acceptor C–N, C–C bonds.

The σ system makes some contribution to delocalization, and important contributions to delocalization correspond to donor–acceptor interactions: C1-C2 → C1-C6, C1-C2 → C1-N26, C1-C2 → C6-H11, C1-C6 → C1–C2, C1-C6 → C1-N26, C1-C6 → C6-H11, C1-N26 → C1-C2, C1-N26 → C2-C3, C2-C3 → C1-C2, C2-C3 → C3-H8, C2-N27 → C1-C2, C2-N27 → C7-H12, C2-N27 → C7-N27, C3-C4 → C2-C3, C3-C4 → C2-N27, C3-H8 → C1-C2, C7-N26 → C1-C6, C7-N27 → C2-C3.

According to Table S2, the delocalization of the σ-electron from σ (C1-C2) is distributed into the antibonding σ* (C1-C6), σ* (C1-N26), and σ* (C6-H11), which leads to stabilization energies of 4.63, 0.86, and 2.42 KJ/mol, respectively. Delocalization of the π-electron from π (C1-C2) is distributed into antibonding π* (C3-C4) and π* (C7-N27), which leads to high stabilization energies of 18.89 and 14.45 KJ/mol, respectively. The delocalization of the σ-electron from σ (C1-C6) is distributed into the antibonding σ* (C1-C2), σ* (C1-N26), and σ* (C6-H11), which leads to stabilization energies of 4.57, 2.82, and 1.04 KJ/mol, respectively. A strong interaction is observed due to the transfer of electron density from the lone pair LP (1) to the antibonding orbitals π* (C1-C2) and π* (C7-N27) with high stabilization energies of 35.08 and 50.10 KJ/mol, respectively.

The values of the polarization coefficients determine the bond formation of the two hybrids. The value of the differences in the polarization coefficients of the atoms (C-O, C-N, C-H bonds) is equal to the differences in the electronegativity of the atoms involved in the bond formation [74]. As can be seen from Table S3, all of the σ(C1-N26), σ(C2-N27), σ(C7-N26), and σ(C7-N27) bond orbitals are polarized towards the nitrogen atoms ED_A_ (%) and ED_B_ (%) with percentage electron densities of about 62.45%, 58.02%, 63.96%, and 58.70% respectively. The σ(C22-H24) orbital with high occupancy 1.98933 a.u. has 60.09% (C22) character in an sp 3.25 hybrid and 39.91% (H24) character in an sp 0.04 hybrid. According to Table S3, it is clear that the natural hybrid orbital LP (1) (N27) having high occupancy (1.92917 a.u.) and low energy (−0.37999 a.u.) has a p-character (0.052%), whereas LP (1) (N26) occupies a high energy orbital (−0.26555 a.u) with p-character (69.99%) and low occupation number (1.57449 a.u).

### 2.7. FTIR Analysis

**Figure 9:**
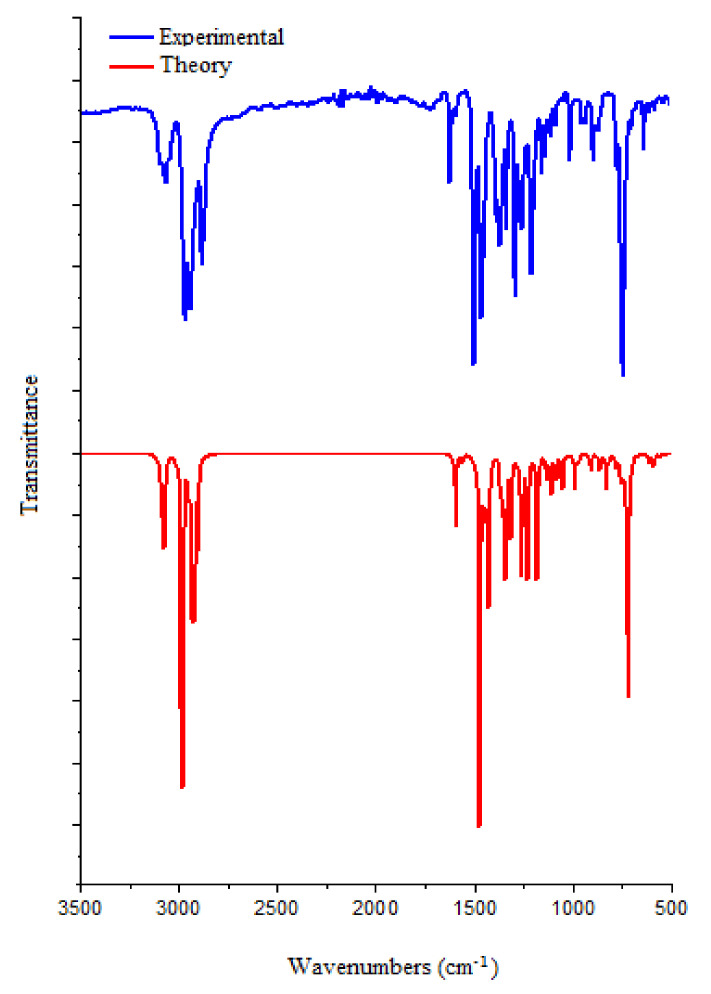
FTIR spectra of the title compound (blue line: experimental, red line: theoretical spectrum).

The density functional theory helps to theoretically establish various physicochemical characteristics of substances [22,75], including spectral data [76,77]. This is important for the structural characterization of substances. The theoretical and experimental FTIR spectra of N-Butyl-1H-benzimidazole are shown in Figure 9, and the ratios of absorption bands are presented in Table S4.

**C-H group vibration**

The predicted stretching vibrations of the C-H group are observed in the range 3110–2900 cm^−1^ at 3110, 3089, 3080, 3070, 3059, 2987, 2983, 2979, 2953, 2936, 2926, 2923, 2919, and 2904 cm^−1^. The CH_2_ scissor mode is assumed to be in the range 1463–1440 cm^−1^. Moreover, vibrations of the C-H group are observed at 1362, 1287, 1251, 1136, 1114, 1092, 993, 943, 882, and 820 cm^−1^. The results obtained agree with the works [76,77].

**C-C group vibration**

The bands observed in the range 1650–1400 cm^−1^ are usually attributed to simple C–C bonding modes for benzene derivatives. In our case, in the calculated C-C spectra, stretching vibrations are observed at 1597, 1563, 1092, 1023, 882, and 759 cm^−1^. Bending vibrations of the C-C group are observed at 1430, 1362, 1272, 1231, 1184, 1054, and 866 cm^−1^. Torsion vibrations of the C-C group in the calculated spectra are observed at 1335, 1251, 1092, 943, 914, 908, 832, 820, and 723 cm^−1^.

**C-N group vibration**

Bands observed closer to 1500 cm^−1^ indicate a C=N double bond, while bands closer to 1300 cm^−1^ indicate the presence of C-N bonds [78]. It is known [19] that vibrations of the C=N group of the imidazole group in benzimidazole are observed at 1490 cm^−1^. In our case, in the calculated spectra, C-N stretching vibrations are observed at 1477, 1362, 1344, 1264, and 1084 cm^−1^. Bending vibrations of the C-N group are observed at 1430, 1054, 832, and 759 cm^−1^. Torsion vibrations of the C-N group in the calculated spectra are observed at 1354, 1335, 832, and 617 cm^−1^.

### 2.8. Mulliken Atomic Charges Analysis

Mulliken atomic charges, calculated by determining the electron population of each atom, play an important role in the application of quantum mechanical calculations and in relation to the vibrational properties of the molecule. Moreover, these charges affect many properties of molecular systems, various aspects of electronic structure, the atomic charge effect, and molecular polarizability [45,79]. Mulliken atomic charges of N-Butyl-1H-benzimidazole calculated at the same basis set are listed in Table S5. According to Table S5, there are negative values on C2-C6 and C13, C19, C22, and N27 atoms, while there are positive values on C1, C7, C16, N26, and hydrogen atoms.

## 3. Materials and Methods

The compound N-Butyl-1H-benzimidazole was purchased from Sigma-Aldrich chemical company.

FTIR spectrum of N-Butyl-1H-benzimidazolewas recorded on Thermo Scientific Nicolet iS10 FTIR spectrophotometer. UV spectra of the N-Butyl-1H-benzimidazole solution (THF solvent) is recorded in 200–600 nm range by using Thermo Scientific UV-Vis Spectrophotometer.

Theoretical calculations were performed using the Gaussian 09 [80] and GaussView 5.0 [81] software packages, and by using Becke’s three parameter exact exchange functional (B3) [82] and three parameter hybrid exchange functional with Lee–Yang–Parr correlation functional [83] method with 6-311++G(d,p) basis set.

The wavefunctions obtained at DFT with 6-311++G(d,p) level were used to determine the electron density ρ_c_ and the Laplace electron density (∇^2^ρ_c_) at the bond critical points (BCPs). All wavefunction analysis was performed by Multiwfn 3.8 program [34]. Multiwfn program was used for topological analysis and to draw electron localization function diagram (ELF) using atom in molecule theory.

## 4. Conclusions

In this work, a comprehensive (theoretical and experimental) study of N-butyl-1H-benzimidazole was carried out. It was shown that the presence of a butyl substituent in the N position has no significant effect on the conjugation and structural organization of benzimidazole and has almost no effect on the C-N bond lengths and C-N-C and N-C-N angles. The nucleophilic and electrophilic regions of N-butyl-1H-benzimidazole were characterized by MEP analysis, Fukui function, and Mulliken atomic charge analysis. It was found that the H atoms and their environment are the most electrophilic centers. FMO (HOMO-LUMO) was useful for evaluating the reactivity of the molecule under study. The value of the coefficients obtained using the NBO analysis shows the contribution of the hybrids to bond formation. The stabilization of the structure of N-Butyl-1H-benzimidazole is evidenced by the strong intramolecular interaction of σ- and π-electrons of donor bonds C-N, C-C with acceptor bonds C-N, C-C. In addition, N-Butyl-1H-benzimidazole was analyzed by AIM, RDG, ELF, and LOL topological methods. The color shades of the ELF and LOL maps confirm the presence of bonding and non-bonding electrons in N-butyl-1H-benzimidazole.

## Figures and Tables

**Table 1:** The theoretical and experimental optimized structural parameters of N-Butyl-1H-benzimidazole.

	B3LYP/6-311++G(d,p)				
	Bond Lengths (A°)		Bond Angles (°)		
Atom Position	Theo.	Exp.	Atom Position	Theo.	Exp.
C_1_-C_2_	1.415	1.398(2)	C_2_-C_1_-C_6_	122.3	107.0
C_1_-C_6_	1.396	1.390(3)	C_2_-C_1_-N_26_	105.1	107.6
C_1_-N_26_	1.386	1.391(2)	C_6_-C_1_-N_26_	132.6	107.5
C_2_-C_3_	1.399	1.371(3)	C_1_-C_2_-C_3_	119.9	107.6
C_2_-N_27_	1.387	1.367(2)	C_1_-C_3_-N_27_	110.1	107.6
C_3_-C_4_	1.389	1.393(3)	C_3_-C_2_-N_27_	130.0	118.9(2)
C_3_-H_8_	1.084	0.930	C_2_-C_3_-C_4_	118.1	107.7(2)
C_4_-C_5_	1.408	1.368(3)	C_2_-C_3_-H_8_	120.2	124(1)
C_4_-H_9_	1.084	0.930	C_4_-C_3_-H_8_	121.7	128(1)
C_5_-C_6_	1.391	1.371(3)	C_3_-C_4_-C_5_	121.4	119.8(2)
C_5_-H_10_	1.084	0.930	C_3_-C_4_-H_9_	119.6	109.4(2)
C_6_-H_11_	1.084	0.930	H_9_-C_4_-C_9_	119.0	130.8(2)
C_7_-H_12_	1.082	0.80(2)	C_4_-C_5_-C_6_	121.5	105.5(2)
C_7_-N_26_	1.377	1.365(3)	C_4_-C_5_-H_10_	119.2	132.3(2)
C_7_-N_27_	1.306	1.317(2)	C_6_-C_5_-H_10_	119.2	122.2(2)
C_13_-H_14_	1.094	0.970	C_1_-C_6_-C_5_	116.9	121.0
C_13_-H_15_	1.095	0.970	C_1_-C_6_-H_11_	122.2	118.0(2)
C_13_-C_16_	1.534	1.449(4)	C_5_-C_6_-H_12_	120.9	121.0
C_13_-N_26_	1.458	-	H_12_-C_7_-N_26_	120.7	121.5
C_16_-H_17_	1.096	0.969	H_12_-C_7_-N_27_	125.0	116.9(2)
C_16_-H_18_	1.095	0.970	N_26_-C_7_-N_27_	114.3	121.6
C_16_-C_19_	1.533	1.510(4)	H_14_-C_13_-H_15_	106.7	119.2
C_19_-H_20_	1.097	0.970	H_14_-C_13_-C_16_	110.2	121.6(2)
C_19_-H_21_	1.097	0.971	C_14_-C_13_-N_26_	107.4	119.2
C_19_-C_22_	1.531	1.417(5)	H_15_-C_13_-C_16_	110.5	121.5(2)
C_22_-H_23_	1.094	0.960	H_15_-C_13_-N_26_	108.2	119.2
C_22_-H_24_	1.093	0.960	C_16_-C_13_-N_26_	113.6	119.3
C_22_-H_25_	1.094	0.959	C_13_-C_16_-H_17_	108.8	123.1(2)
			C_13_-C_16_-H_18_	109.3	124.8(2)
			C_13_-C_16_-C_19_	112.4	112.1(2)
			H_17_-C_16_-H_18_	106.4	108.3
			H_17_-C_16_-C_19_	109.9	108.3
			H_18_-C_16_-C_19_	109.8	115.7(2)
			C_16_-C_19_-H_20_	109.4	107.5
			C_16_-C_19_-H_21_	109.4	108.4
			C_16_-C_19_-C_22_	112.9	108.3
			H_20_-C_19_-H_21_	106.1	107.8
			H_20_-C_19_-C_22_	109.4	107.9
			H_21_-C_19_-C_22_	109.4	117.8(3)
			C_19_-C_22_-H_23_	111.2	107.3
			C_19_-C_22_-H_24_	111.3	107.9
			C_19_-C_22_-H_25_	111.2	107.8
			H_23_-C_22_-H_24_	107.7	109.5
			H_23_-C_22_-H_25_	107.6	109.5
			H_24_-C_22_-H_25_	107.7	109.5
			C_1_-N_26_-C_7_	105.9	109.5
			C_1_-N_26_-C_13_	127.4	109.4
			C_7_-N_26_-C_13_	126.7	109.4
			C_2_-N_27_-C_7_	104.6	105.4(2)

**Table 2:** AIM topological parameters at BCPs (ρ: electron density; ∇^2^ρ: Laplacian of electron density; H: total energy density; V: potential energy density, and E_interactions_: interaction energy).

Interactions Types	∇^2^ρ(r) (a.u.)	ρ (r) (a.u.)	G(r) (a.u.)	V(r) (a.u.)	H(r) (a.u.)	ε	E_interactions_ kJ/mol
**RCP1**	0.0140	0.0040	0.0029	−0.0023	0.0006	−2.0003	−3.02
**RCP2**	−0.0176	0.3888	0.3888	−0.6782	−0.5589	0.0041	−890.05
**RCP3**	0.0071	0.0018	0.0013	−0.0009	0.0005	−1.9466	−1.18
**RCP4**	0.1673	0.0226	0.0342	−0.0265	0.0077	−1.2088	−34.13
**NRCP1**	0.3993	0.0560	0.0939	−0.0879	0.0059	−1.2860	−112.89
**NRCP2**	0.3980	0.0558	0.0936	−0.0876	0.0059	1.2847	−112.89
**NRCP3**	0.1671	0.0225	0.0341	−0.0265	0.0077	−1.2076	−34.13
**C_43_-H_45_…N_26_**	0.0123	0.0033	0.0026	−0.0022	0.0005	0.4900	−28.88
**N_31_-H_54_…N_26_**	0.0874	0.0211	0.0182	−0.0145	0.0037	0.0298	−18.90
**C_7_-H_8_…H_16_**	0.0069	0.0019	0.0013	−0.0009	0.0004	1.8492	−1.05
**C_36_-H_37_…H_2_**	0.0063	0.0017	0.0012	−0.0008	0.0004	2.9057	−1.05

**Table 3:** HOMO-LUMO energy and other electronic properties of title compound.

Quantum Parameters	DFT/B3LYP/6-31++G(d, p)
E_HOMO_(eV)	−6.26
E_LUMO_(eV)	−0.82
E_HOMO+1_(eV)	−6.37
E_LUMO-1_ (eV)	−0.81
│ΔE_HOMO-LUMO_│ (eV)	5.44
│ΔE_HOMO+1-LUMO-1_│ (eV)	5.56
I	6.26
A	0.82
χ	3.39
η	2.72
µ	−3.39
ω	2.11
S	0.18

ΔE = │E_HOMO_ − E_LUMO_│(eV), I = −E_HOMO_ (eV), A = −E_LUMO_, χ = (I + A)/2, η = (I − A)/2, µ = −(I + A)/2, ω = µ^2^/2 η, S = 1/2 η.

## Data Availability

Not applicable.

## Supplementary Materials

The following supporting information can be downloaded at: https://www.mdpi.com/article/10.3390/molecules27227864/s1, Table S1: Condensed Fukui functions for NBB; Table S2: Second order perturbation theory analysis of Fock matrix in NBO basis for N-Butyl-1H-benzimidazole; Table S3: Calculated natural bond orbitals (NBO) and the polarization coefficient for each hybrid in selected bonds of the C1 using the B3LYP/6-311++G(d,p) in the gas phase for NBB; Table S4: Theoretical wavenumber (cm^−1^) of N-Butyl-1H-benzimidazole calculated by means of VEDA 4 program; Table S5: Mulliken atomic charges of N-Butyl-1H-benzimidazole.

## Sample Availability

Samples of the compounds are available from the authors.

## References

[1] Pathare B., Bansode T. (2021). Review- biological active benzimidazole derivatives. Results Chem..

[2] Bansal Y., Silakari O. (2012). The therapeutic journey of benzimidazoles: A review. Bioorganic Med. Chem..

[3] Cheng J., Xie J., Luo X. (2005). Synthesis and antiviral activity against Coxsackie virus B3 of some novel benzimidazole derivatives. Bioorganic Med. Chem. Lett..

[4] Charlson A.J. (1973). The methanesulfonylation of 2-benzimidazolemethanol and α(2-benzimidazolyl)benzyl alcohol. Carbohydr. Res..

[5] Walker K.A.M., Braemer A.C., Hitt S., Jones R.E., Matthews T.R. (1978). 1-[4-(4-Chlorophenyl)-2-(2,6-dichlorophenylthio)-n-butyl]-1H-imidazole nitrate, a new potent antifungal agent. J. Med. Chem..

[6] Sheng J., Nguyen P.T.M., Baldeck J.D., Olsson J., Marquis R.E. (2006). Antimicrobial actions of benzimidazoles against the oral anaerobes Fusobacterium nucleatum and Prevotella intermedia. Arch. Oral Biol..

[7] Infante-Castillo R., Rivera-Montalvo L.A., Hernández-Rivera S.P. (2008). Theoretical DFT, vibrational and NMR studies of benzimidazole and alkyl derivatives. J. Mol. Struct..

[8] Drolet D.P., Manuta D.M., Lees A.J., Katnani A.D., Coyle G.J. (1988). FT-IR and XPS study of copper(II) complexes of imidazole and benzimidazole. Inorg. Chim. Acta.

[9] Shaharyar M., Mazumder A. (2017). Benzimidazoles: A biologically active compounds. Arab. J. Chem..

[10] Shannon M.S., Hindman M.S., Danielsen S.P.O., Tedstone J.M., Gilmore R.D., Bara J.E. (2012). Properties of alkylbenzimidazoles for CO2 and SO2 capture and comparisons to ionic liquids. Sci. China Chem..

[11] Chen S.-H., Zhao Q., Xu X.-W. (2008). Preparation and characterization of a novel benzimidazolium brønsted acidic ionic liquid and its application in esterifications. J. Chem. Sci..

[12] Veerasamy R., Roy A., Karunakaran R., Rajak H. (2021). Structure–Activity Relationship Analysis of Benzimidazoles as Emerging Anti-Inflammatory Agents: An Overview. Pharmaceuticals.

[13] Rutkowska I.A., Marszalek M., Orlowska J., Ozimek W., Zakeeruddin S.M., Kulesza P.J., Grätzel M. (2015). Nanocomposite Semi-Solid Redox Ionic Liquid Electrolytes with Enhanced Charge-Transport Capabilities for Dye-Sensitized Solar Cells. ChemSusChem.

[14] Chen J.-H., Ahmed W., Li M.-H., Li Z.-D., Cui Z.-N., Tang R.-Y. (2020). TEMPO-Mediated Synthesis of N-(Fluoroalkyl)imidazolones via Reaction of Imidazoles with Iodofluoroacetate. Adv. Synth. Catal..

[15] Hussaini S.Y., Haque R.A., Fatima T., Agha T.M., Abdul Majid A.M.S., Abdallah H.H., Razali M.R. (2018). Nitrile functionalized silver(I) N-heterocyclic carbene complexes: DFT calculations and antitumor studies. Transit. Met. Chem..

[16] Wu H., Jin C., Huang G., Wang L., Jiang J., Wang L. (2011). Binaphthyl-bridged bis-imidazolinium salts as N-heterocyclic carbene ligand precursors in the palladium-catalyzed Heck reaction. Sci. China Chem..

[17] Shukla M., Saha S. (2013). Relationship between stabilization energy and thermophysical properties of different imidazolium ionic liquids: DFT studies. Comput. Theor. Chem..

[18] Dzyuba S.V., Bartsch R.A. (2001). New room-temperature ionic liquids with -symmetrical imidazolium cations. Chem. Commun..

[19] Aman H., Rashid N., Ashraf Z., Bibi A., Chen H.-T., Sathishkumar N. (2020). Synthesis, density functional theory (DFT) studies and urease inhibition activity of chiral benzimidazoles. Heliyon.

[20] Halls M.D., Velkovski J., Schlegel H.B. (2001). Harmonic frequency scaling factors for Hartree-Fock, S-VWN, B-LYP, B3-LYP, B3-PW91 and MP2 with the Sadlej pVTZ electric property basis set. Theor. Chem. Acc..

[21] Kazachenko A.S., Akman F., Malyar Y.N., Issaoui N., Vasilieva N.Y., Karacharov A.A. (2021). Synthesis optimization, DFT and physicochemical study of chitosan sulfates. J. Mol. Struct..

[22] Kazachenko A.S., Tomilin F.N., Pozdnyakova A.A., Vasilyeva N.Y., Malyar Y.N., Kuznetsova S.A., Avramov P.V. (2020). Theoretical DFT interpretation of infrared spectra of biologically active arabinogalactan sulphated derivatives. Chem. Pap..

[23] Brown I.D. (2002). Topology and Chemistry. Struct. Chem..

[24] Bader R.F.W. (1990). Atoms in Molecules A Quantum Theory.

[25] Bader R.F.W. (1989). Atoms in molecules in external fields. J. Chem. Phys..

[26] Akman F., Issaoui N., Kazachenko A.S. (2020). Intermolecular hydrogen bond interactions in the thiourea/water complexes (Thio-(H2O)n) (n = 1, …, 5): X-ray, DFT, NBO, AIM, and RDG analyses. J. Mol. Model..

[27] Kazachenko A., Akman F., Medimagh M., Issaoui N., Vasilieva N., Malyar Y.N., Sudakova I.G., Karacharov A., Miroshnikova A., Al-Dossary O.M. (2021). Sulfation of Diethylaminoethyl-Cellulose: QTAIM Topological Analysis and Experimental and DFT Studies of the Properties. ACS Omega.

[28] Kazachenko A.S., Akman F., Abdelmoulahi H., Issaoui N., Malyar Y.N., Al-Dossary O., Wojcik M.J. (2021). Intermolecular hydrogen bonds interactions in water clusters of ammonium sulfamate: FTIR, X-ray diffraction, AIM, DFT, RDG, ELF, NBO analysis. J. Mol. Liq..

[29] Medimagh M., Issaoui N., Gatfaoui S., Al-Dossary O.S., Kazachenko A., Marouani H., Wojcik M.J. (2021). Molecular modeling and biological activity analysis of new organic-inorganic hybrid: 2-(3,4-dihydroxyphenyl) ethanaminium nitrate. J. King Saud Univ.-Sci..

[30] Domingo L.R., Pérez P. (2014). A quantum chemical topological analysis of the C–C bond formation in organic reactions involving cationic species. Phys. Chem. Chem. Phys..

[31] Fuster F., Sevin A., Silvi B. (2000). Topological Analysis of the Electron Localization Function (ELF) Applied to the Electrophilic Aromatic Substitution. J. Phys. Chem. A.

[32] Hernández-Trujillo J., García-Cruz I., Martínez-Magadán J.M. (2005). Topological analysis of the electron density and of the electron localization function of pyrene and its radicals. Chem. Phys..

[33] Maulén B., Echeverri A., Gómez T., Fuentealba P., Cárdenas C. (2019). Electron Localization Function in Excited States: The Case of the Ultrafast Proton Transfer of the Salicylidene Methylamine. J. Chem. Theory Comput..

[34] Lu T., Chen F. (2012). Multiwfn: A multifunctional wavefunction analyzer. J. Comput. Chem..

[35] Vincy C.D., Tarika J.D.D., Dexlin X.D.D., Rathika A., Beaula T.J. (2022). Exploring the antibacterial activity of 1, 2 diaminoethane hexanedionic acid by spectroscopic, electronic, ELF, LOL, RDG analysis and molecular docking studies using DFT method. J. Mol. Struct..

[36] Stewart J.J.P. (2018). An examination of the nature of localized molecular orbitals and their value in understanding various phenomena that occur in organic chemistry. J. Mol. Model..

[37] Jacobsen H. (2008). Localized-orbital locator (LOL) profiles of chemical bonding. Can. J. Chem..

[38] Saleh G., Gatti C., Lo Presti L. (2012). Non-covalent interaction via the reduced density gradient: Independent atom model vs experimental multipolar electron densities. Comput. Theor. Chem..

[39] Silvi B., Savin A. (1994). Classification of chemical bonds based on topological analysis of electron localization functions. Nature.

[40] Gadre S.R., Suresh C.H., Mohan N. (2021). Electrostatic Potential Topology for Probing Molecular Structure, Bonding and Reactivity. Molecules.

[41] Johnson E.R., Keinan S., Mori-Sánchez P., Contreras-García J., Cohen A.J., Yang W. (2010). Revealing Noncovalent Interactions. J. Am. Chem. Soc..

[42] Sevvanthi S., Muthu S., Raja M. (2018). Molecular docking, vibrational spectroscopy studies of (RS)-2-(tert-butylamino)-1-(3-chlorophenyl)propan-1-one: A potential adrenaline uptake inhibitor. J. Mol. Struct..

[43] Kazachenko A.S., Akman F., Sagaama A., Issaoui N., Malyar Y.N., Vasilieva N.Y., Borovkova V.S. (2021). Theoretical and experimental study of guar gum sulfation. J. Mol. Model..

[44] Barim E., Akman F. (2019). Synthesis, characterization and spectroscopic investigation of N-(2-acetylbenzofuran-3-yl)acrylamide monomer: Molecular structure, HOMO–LUMO study, TD-DFT and MEP analysis. J. Mol. Struct..

[45] Akman F., Kazachenko A., Malyar Y. (2021). A density functional theory study of sulfated monolignols: P-Coumaril and coniferyl alcohols. Cellul. Chem. Technol..

[46] Noureddine O., Issaoui N., Medimagh M., Al-Dossary O., Marouani H. (2021). Quantum chemical studies on molecular structure, AIM, ELF, RDG and antiviral activities of hybrid hydroxychloroquine in the treatment of COVID-19: Molecular docking and DFT calculations. J. King Saud Univ.-Sci..

[47] Medimagh M., Issaoui N., Gatfaoui S., Antonia Brandán S., Al-Dossary O., Marouani H.J., Wojcik M. (2021). Impact of non-covalent interactions on FT-IR spectrum and properties of 4-methylbenzylammonium nitrate. A DFT and molecular docking study. Heliyon.

[48] Almeida M.O., Barros D.A.S., Araujo S.C., Faria S.H.D.M., Maltarollo V.G., Honorio K.M. (2017). Study on molecular structure, spectroscopic properties (FTIR and UV–Vis), NBO, QTAIM, HOMO-LUMO energies and docking studies of 5-fluorouracil, a substance used to treat cancer. Spectrochim. Acta Part A Mol. Biomol. Spectrosc..

[49] Akman F., Kazachenko A.S., Vasilyeva N.Y., Malyar Y.N. (2020). Synthesis and characterization of starch sulfates obtained by the sulfamic acid-urea complex. J. Mol. Struct..

[50] Buvaneswari M., Santhakumari R., Usha C., Jayasree R., Sagadevan S. (2021). Synthesis, growth, structural, spectroscopic, optical, thermal, DFT, HOMO–LUMO, MEP, NBO analysis and thermodynamic properties of vanillin isonicotinic hydrazide single crystal. J. Mol. Struct..

[51] Muthu S., Renuga S. (2014). Vibrational spectra and normal coordinate analysis of 2-hydroxy-3-(2-methoxyphenoxy) propyl carbamate. Spectrochim. Acta Part A Mol. Biomol. Spectrosc..

[52] Parr R.G., Donnelly R.A., Levy M., Palke W.E. (1978). Electronegativity: The density functional viewpoint. J. Chem. Phys..

[53] Alameen A.A., Abdalla M., Alshibl H.M., AlOthman M.R., Alkhulaifi M.M., Mirgany T.O., Elsayim R. (2022). In-silico studies of glutathione peroxidase4 activators as candidate for multiple sclerosis management. J. Saudi Chem. Soc..

[54] Pineda L.H., Tecuapa-Flores E.D., Hernández J.G., Thangarasu P., Vázquez Ramos J.M. (2021). Ruthenium complex of bis(benzimidazole-yl-ethyl)sulfide as chemo-sensor for selective recognition of chloride ion, and its application in real bacterial samples. Inorg. Chim. Acta.

[55] Camacho-Mendoza R.L., Gutiérrez-Moreno E., Guzmán-Percástegui E., Aquino-Torres E., Cruz-Borbolla J., Rodríguez-Ávila J.A., Alvarado-Rodríguez J.G., Olvera-Neria O., Thangarasu P., Medina-Franco J.L. (2015). Density Functional Theory and Electrochemical Studies: Structure–Efficiency Relationship on Corrosion Inhibition. J. Chem. Inf. Model..

[56] Guadalupe H.J., Narayanan J., Pandiyan T. (2011). Synthesis, molecular structure and spectral analysis: DFT–TDDFT computational study of ruthenium complex of tetradentate N,N′-bis(benzimidazole-2yl-ethyl)-ethylenediamine. J. Mol. Struct..

[57] Najiya A., Panicker C.Y., Sapnakumari M., Narayana B., Sarojini B.K., Van Alsenoy C. (2014). Molecular structure, FT-IR, first order hyperpolarizability, NBO analysis, HOMO and LUMO, MEP analysis of (E)-3-(4-chlorophenyl)-1-(4-fluorophenyl)prop-2-en-1-one by HF and density functional methods. Spectrochim. Acta Part A Mol. Biomol. Spectrosc..

[58] Lutoshkin M.A., Petrov A.I., Kuznetsov B.N., Kazachenko A.S. (2019). Aqueous Complexation of Morin and Its Sulfonate Derivative with Lanthanum(III) and Trivalent Lanthanides. J. Solut. Chem..

[59] Parr R.G., Yang W. (1989). Density Functional Theory of Atoms and Molecules.

[60] Geerlings P., De Proft F., Langenaeker W. (2003). Conceptual Density Functional Theory. Chem. Rev..

[61] Chattaraj P.K., Sarkar U., Toro-Labbé A. (2007). Chapter 13 Chemical reactivity dynamics in ground and excited electronic states. Theoretical and Computational Chemistry.

[62] Kolandaivel P., Praveena G., Selvarengan P. (2005). Study of atomic and condensed atomic indices for reactive sites of molecules. J. Chem. Sci..

[63] Raja M., Raj Muhamed R., Muthu S., Suresh M., Muthu K. (2017). Synthesis, spectroscopic (FT-IR, FT-Raman, NMR, UV–Visible), Fukui function, antimicrobial and molecular docking study of (E)-1-(3-bromobenzylidene)semicarbazide by DFT method. J. Mol. Struct..

[64] Çankaya N., Tanış E. (2018). Synthesis, characterization and in-silico estimation of the toxic potential of *N*-(4-nitrophenyl)methacrylamide. Mater. Res. Express.

[65] Sheeba B.Q., Michael Mary M.S., Amalanathan M., Job C.B. (2021). Structural and vibrational spectral investigation on the identification of Non-Linear Optical properties and wave function analyses (electrostatic potential, electron localisation function, localised orbital locator) of 3-Ethoxy Salicilaldehyde. Mol. Simul..

[66] Lutoshkin M.A., Kazachenko A.S. (2017). Assessment of various density functionals and solvation models to describe acid-base, spectral and complexing properties of thiobarbituric and barbituric acids in aqueous solution. J. Comput. Methods Sci. Eng..

[67] Ahmad M.S., Khalid M., Shaheen M.A., Tahir M.N., Khan M.U., Braga A.A.C., Shad H.A. (2018). Synthesis and XRD, FT-IR vibrational, UV–vis, and nonlinear optical exploration of novel tetra substituted imidazole derivatives: A synergistic experimental-computational analysis. J. Phys. Chem. Solids.

[68] Tahir M.N., Khalid M., Islam A., Ali Mashhadi S.M., Braga A.A.C. (2017). Facile synthesis, single crystal analysis, and computational studies of sulfanilamide derivatives. J. Mol. Struct..

[69] Weinhold F., Landis C.R., Glendening E.D. (2016). What is NBO analysis and how is it useful?. Int. Rev. Phys. Chem..

[70] Sagaama A., Issaoui N., Al-Dossary O., Kazachenko A.S., Wojcik M.J. (2021). Non covalent interactions and molecular docking studies on morphine compound. J. King Saud Univ.-Sci..

[71] Jayabharathi J., Thanikachalam V., Jayamoorthy K. (2013). Optical properties of 1,2-diaryl benzimidazole derivatives – A combined experimental and theoretical studies. Spectrochim. Acta Part A Mol. Biomol. Spectrosc..

[72] Lutoshkin M.A., Petrov A.I., Malyar Y.N., Kazachenko A.S. (2021). Interaction of Rare-Earth Metals and Some Perfluorinated β-Diketones. Inorg. Chem..

[73] Akman F. (2021). A comparative study based on molecular structure, spectroscopic, electronic, thermodynamic and NBO analysis of some nitrogen-containing monomers. Polym. Bull..

[74] Ouaket A., Chraka A., Raissouni I., Amrani M.A.E., Berrada M., Knouzi N. (2022). Synthesis, spectroscopic (13C/1H-NMR, FT-IR) investigations, quantum chemical modelling (FMO, MEP, NBO analysis), and antioxidant activity of the bis-benzimidazole molecule. J. Mol. Struct..

[75] Profant V., Johannessen C., Blanch E.W., Bouř P., Baumruk V. (2019). Effects of sulfation and the environment on the structure of chondroitin sulfate studied via Raman optical activity. Phys. Chem. Chem. Phys..

[76] Akman F. (2016). Spectroscopic investigation, HOMO–LUMO energies, natural bond orbital (NBO) analysis and thermodynamic properties of two-armed macroinitiator containing coumarin with DFT quantum chemical calculations. Can. J. Phys..

[77] Kazachenko A.S., Malyar Y.N., Ghatfaoui S., Issaoui N., Al-Dossary O., Wojcik M.J., Kazachenko A.S., Miroshnikova A.V., Berezhnaya Y.D. (2022). A density functional theory calculations of infrared spectra of galactomannan butyl ether. J. Mol. Struct..

[78] Majoube M. (1984). Vibrational spectra of guanine. A normal coordinate analysis. J. Mol. Struct..

[79] Mulliken R.S. (1955). Electronic Population Analysis on LCAO–MO Molecular Wave Functions. I. J. Chem. Phys..

[80] Frisch M.J., Trucks G.W., Schlegel H.B., Scuseria G.E., Robb M.A., Cheeseman J.R., Scalmani G., Barone V., Mennucci B., Petersson G.A. (2009). Gaussian 09, Revision C.01.

[81] Guassian, Inc GaussView.

[82] Becke A.D. (1988). Density-functional exchange-energy approximation with correct asymptotic behavior. Phys. Rev. A.

[83] Lee C., Yang W., Parr R.G. (1988). Development of the Colle-Salvetti correlation-energy formula into a functional of the electron density. Phys. Rev. B.

