# Supplementary material for: A Comprehensive Study of N-Butyl-1H-Benzimidazole
Source: Molecules. 2022 Nov 14;27(22):7864. doi: 10.3390/molecules27227864 (PMC9698437; doi:10.3390/molecules27227864)
Supplement: Supplementary file 1 [file molecules-27-07864-s001.zip › molecules-2033937-supplementary.pdf]

A Comprehensive Study of N-Butyl-1H-Benzimidazole

Aleksandr S. Kazachenko [1,2,3,*], Emine Tanış [4], Feride Akman [5,*], Mouna Medimagh [6], Noureddine Issaoui [6], Omar Al-Dossary [7], Leda G. Bousiakou [8], Anna S. Kazachenko [1,2], Dmitry Zimonin [1,2] and Andrey M. Skripnikov [1,2]

1 School of Non-Ferrous Metals and Material Science, Siberian Federal University, Pr. Svobodny 79, 660041 Krasnoyarsk, Russia
2 Institute of Chemistry and Chemical Technology, Krasnoyarsk Scientific Center, Siberian Branch, Russian Academy of Sciences, Akademgorodok, 50, Bld. 24, 660036 Krasnoyarsk, Russia
3 Department of Biological Chemistry with Courses in Medical, Pharmaceutical and Toxicological Chemistry, Krasnoyarsk State Medical University of the Ministry of Healthcare of the Russian Federation, St. Partizan Zheleznyak, Bld. 1, 660022 Krasnoyarsk, Russia
4 Department of Electrical Electronics Engineering, Faculty of Engineering and Architecture, Kırşehir Ahi Evran University, Kırşehir 40100, Turkey
5 Vocational School of Food, Agriculture and Livestock, University of Bingöl, Bingöl 12000, Turkey
6 Laboratory of Quantum and Statistical Physics (LR18ES18), Faculty of Sciences, University of Monastir, Monastir 5000, Tunisia
7 Department of Physics and Astronomy, College of Science, King Saud University, P.O. Box 2455, Riyadh 11451, Saudi Arabia
8 IMD Laboratories Co., R&D Section, Lefkippos Technology Park, NCSR Demokritos, P.O. Box 60037, 15130 Athens, Greece
* Correspondence: leo_lion_leo@mail.ru (A.S.K.); chemakman@gmail.com (F.A.)

Supplementary Materials

Table S1. Condensed Fukui functions for NBB

**Atoms**	f_r^+	f_r^-	f_r^0	Δf(r)
C1	-18,8264	2,3029	-8,2618	-21,1293
C2	13,0512	-7,4194	2,8159	20,4706
C3	-6,3062	-3,0305	-4,6684	-3,2757
C4	11,3780	-5,7455	2,8163	17,1235
C5	12,1912	-5,1466	3,5223	17,3378
C6	2,1966	-4,0100	-0,9067	6,2066
C7	-15,9949	-7,7970	-11,8960	-8,1979
H8	-5,3944	-6,3091	-5,8518	0,9147
H9	-4,3771	-7,4156	-5,8964	3,0385
H10	-10,0579	-7,0430	-8,5505	-3,0149
H11	-30,9569	-5,8619	-18,4094	-25,0950
H12	-31,7882	-6,9532	-19,3707	-24,8350

C13	44,7673	1,0525	22,9099	43,7148
H14	-26,8419	-3,3110	-15,0765	-23,5309
H15	4,8414	-3,0603	0,8905	7,9017
C16	8,3801	2,5912	5,4857	5,7889
H17	31,6032	-0,4020	15,6006	32,0052
H18	-14,5065	-2,2840	-8,3953	-12,2225
C19	6,0125	1,0261	3,5193	4,9864
H20	-40,8605	-0,8910	-20,8758	-39,9695
H21	-46,2341	-0,9343	-23,5842	-45,2998
C22	148,0819	-3,1314	72,4753	151,2133
H23	-37,8624	-0,2530	-19,0577	-37,6094
H24	-57,6031	-1,9546	-29,7789	-55,6485
H25	-23,4681	-0,7722	-12,1202	-22,6959
N26	-3,0917	-3,8957	-3,4937	0,8040
N27	-6,1914	-9,4666	-7,8290	3,2752

Table S2. Second order perturbation theory analysis of Fock matrix in NBO basis for N-Butyl-1H-benzimidazole.

Donor (i)	Type	ED/e	Acceptor(j)	Type	ED/e	$E^{(2)a}$(KJ mol^{-1})	E(j)-E(i)^{b} (a.u)	F(i.j)^{c} (a.u)
C1-C2	σ	1.96	C1-C6	σ*	0.03	4.63	1.24	0.068
C1-C2	σ	1.96	C1-N26	σ*	0.03	0.86	1.12	0.028
C1-C2	σ	1.96	C6-H11	σ*	0.01	2.42	1.12	0.046
C1-C2	π	1.59	C3-C4	π*	0.31	18.89	0.28	0.067
C1-C2	π	1.59	C7-N27	π*	0.35	14.45	0.25	0.055
C1-C6	σ	1.97	C1 –C2	σ*	0.03	4.57	1.25	0.068
C1-C6	σ	1.97	C1-N26	σ*	0.03	2.82	1.15	0.051
C1-C6	σ	1.97	C6-H11	σ*	0.01	1.04	1.15	0.031
C1-N26	σ	1.98	C1-C2	σ*	0.03	1.03	1.34	0.033
C1-N26	σ	1.98	C2-C3	σ*	0.02	2.53	1.37	0.053

C2-C3	σ	1.97	C1-C2	σ*	0.03	3.55	1.24	0.059
C2-C3	σ	1.97	C3-H8	σ*	0.01	0.95	1.15	0.030
C2-N27	σ	1.98	C1-C2	σ*	0.03	1.17	1.31	0.035
C2-N27	σ	1.98	C7-H12	σ*	0.02	5.04	1.18	0.069
C2-N27	σ	1.98	C7-N27	σ*	0.01	0.71	1.34	0.028
C3-C4	σ	1.97	C2-C3	σ*	0.02	3.01	1.27	0.055
C3-C4	σ	1.97	C2-N27	σ*	0.02	5.47	1.17	0.071
C3-C4	π	1.72	C1-C2	π *	0.5	17.97	0.28	0.066
C3-C4	π	1.72	C5-C6	σ*	0.01	19.46	0.28	0.067
C3-H8	σ	1.98	C1-C2	σ*	0.03	4.09	1.06	0.059
C4-C5	π	1.71	C3-C4	π*	0.31	2.62	1.28	0.052
C5-C6	π	1.72	C1-C2	π*	0.47	19.58	0.28	0.070
C5-C6	π	1.72	C3-C4	π*	0.31	17.74	0.29	0.065
C7-N26	σ	1.98	C1-C6	σ*	0.02	4.68	1.37	0.072
C7-N27	σ	1.98	C2-C3	σ*	0.02	5.70	1.42	0.081
C7-N27	π	1.88	C1-C2	π*	0.47	18.41	0.34	0.078
N26	LP(1)	1.57	C1-C2	π*	0.47	35.08	0.29	0.091
N26	LP(1)	1.57	C7-N27	π*	0.35	50.10	0.26	0.105
N27	LP(1)	1.57	C1-C2	π*	0.47	5.88	0.92	0.066
N27	LP(2)	1.83	C7-N26	π*	0.35	7.60	0.81	0.071
C7-N27	π*	1.88	C1-C2	π*	0.47	73.61	0.50	0.061

Table S3. Calculated natural bond orbitals (NBO) and the polarization coefficient for each hybrid in selected bonds of the C1 using the B3LYP/6-311++G(d,p) in the gas phase for NBB.

Occupancy (a.u.)	Bond (A-B)[a]	Energy (a.u.)	ED_A (%)	ED_B (%)	NBO	S(%)(A)	S(%)(B)	P(%) (A)	P(%) (B)
1.96507	σ (C1-C2)	-0.67599	51.00	49.00	0.7142 ($sp^{2.03}$) + 0.7000 ($sp^{2.09}$)	32.94	32.33	67.01	67.62

1.58979	π (C1-C2)	-0.25537	49.97	50.03	0.7069 ($sp^{1.00}$) + 0.7073 ($sp^{1.00}$)	0.00	0.00	99.97	99.97
1.97476	σ (C1-C6)	-0.71441	51.65	48.35	0.7187 ($sp^{1.51}$) +0.6953 ($sp^{1.94}$)	39.85	33.98	60.12	65.97
1.98139	σ(C1-N26)	-0.80177	37.55	62.45	0.6128 ($sp^{2.70}$) +0.7903 ($sp^{2.0}$)	27.03	33.28	72.87	66.67
1.97699	σ(C2-C3)	-0.70264	51.29	48.71	0.7161($sp^{1.62}$) +0.6980 ($sp^{1.90}$)	38.21	34.44	61.76	65.51
1.97730	σ(C2-N27)	-0.77181	41.98	58.02	0.6479 ($sp^{2.42}$) +0.7617 ($sp^{2.10}$)	29.19	32.20	70.71	67.71
1.97685	σ(C3-C4)	-0.70116	50.49	49.51	0.7105 ($sp^{1.75}$) +0.7037 ($sp^{1.78}$)	36.35	35.96	63.61	64.00
1.72398	π(C3-C4)	-0.25652	49.07	50.93	0.7005 ($sp^{1.00}$) +0.7137 ($sp^{1.00}$)	0.00	0.00	99.95	99.96
1.97965	σ(C3-H8)	-0.51968	61.00	39.00	0.7810 ($sp^{2.42}$) +0.6245 ($sp^{0.00}$)	29.21	99.96	70.75	0.04
1.97934	σ(C4-C5)	-0.69084	49.95	50.05	0.7068 ($sp^{1.81}$) +0.7075 ($sp^{1.80}$)	35.54	35.73	64.42	64.23
1.97989	σ(C4-H9)	-0.51316	60.65	39.35	0.7788 ($sp^{2.51}$) +0.6273 ($sp^{0.00}$)	28.52	99.95	71.44	0.05
1.97559	σ(C5-C6)	-0.70186	49.23	50.77	0.7017 ($sp^{1.79}$) +0.7125 ($sp^{1.75}$)	35.79	36.41	64.16	63.55
1.72737	π(C5-C6)	-0.25897	49.34	50.66	0.7017 ($sp^{1.00}$) +0.7125 ($sp^{1.00}$)	0.00	0.00	99.95	99.95
1.97953	σ(C5-H10)	-0.51465	60.67	39.33	0.7789 ($sp^{2.51}$) +0.6272 ($sp^{0.00}$)	28.48	99.95	71.47	0.05

1.97870	σ(C6-H11)	-0.52629	61.22	38.78	0.7824 ($sp^{2.37}$) +0.6272 ($sp^{0.00}$)	29.62	99.95	70.34	0.05
1.98436	σ(C7-H12)	-0.56257	60.57	39.43	0.7782 ($sp^{1.93}$) +0.6280 ($sp^{0.00}$)	34.14	99.94	95.82	0.06
1.98559	σ(C7-N26)	-0.80774	36.04	63.96	0.6004 ($sp^{2.27}$) +0.7997 ($sp^{2.11}$)	30.57	32.14	69.31	67.81
1.98933	σ(C22-H24)	-0.49246	60.09	39.91	0.6004 ($sp^{3.25}$) +0.7997 ($sp^{0.04}$)	23.52	99.96	76.42	0.07
1.98359	σ(C7-N27)	-0.32006	41.30	58.70	0.6426 ($sp^{1.82}$) +0.7662 ($sp^{1.84}$)	35.38	35.38	64.53	64.77
1.97887	σ(C13-C16)	-0.61557	50.81	49.19	0.7128 ($sp^{2.27}$) +0.7014 ($sp^{2.69}$)	30.57	27.08	69.39	72.88
1.57449	LP (1) (N26)	-0.26555	-	-	($sp^{99.99}$)	99.94	-	66.99	-
1.92917	LP (1) (N27)	-0.37999	-	-	($sp^{69.99}$)	99.99	-	0.052	

Table S4. Theoretical wavenumber (cm^{-1}) of N-Butyl-1H-benzimidazole calculated by means of VEDA 4 program

No	Theoretical Wavenumber (cm^{-1})		TED (<10%)	Experimental Wavenumber (cm^{-1})	No	Theoretical Wavenumber (cm^{-1})		TED (<10%)	Experimental Wavenumber (cm^{-1})
	Scaled	I_{IR}	Assignments			Scaled	I_{IR}	Assignments	
1	3110,892	1.60	νCH (99)		37	1136,174	6.91	νCC (11) + δHCC (34)+ δHCC (17)	
2	3089,168	11.15	νCH (63) + νCH (24)	3100	38	1114,614	10.62	δHCC (16)	1080
3	3080,708	18.92	νCH (40) + νCH (34)	3001	39	1092,948	3.23	νCC (14)+ τHCCC (10)	
4	3070,247	11.86	νCH (10) + νCH (44)		40	1084,247	5.04	νNC (11)	
5	3059,912	0.21	νCH (47)		41	1054,498	11.05	δCCC (10) + δNCN (25)	1006
6	2987,045	40.60	νCH (74)		42	1023,222	0.66	νCC (37)	
7	2983,835	75.32	νCH (11) + νCH (23)	2900	43	993,271	8.48	δHCC (13)+ δHCC (11) + δCCC (19)	

8	2979,127	0.41	νCH (31) + νCH (23)		44	976,7484	2.51	νCC (34)	
9	2953,903	4.87	νCH (18) + νCH (37)		45	943,4034	0.06	τHCCC (33) + τHCCC (16)	
10	2936,887	57.30	νCH (28) + νCH (59)		46	914,0031	3.06	τHCCC (13)	
11	2926,823	8.74	νCH (45)		47	908,6566	2.36	τHCCC (27) + τHCCC (35)	
12	2923,439	31.22	νCH (18) + νCH (27)+ νCH (18)+νCH (22)		48	882,1953	0.29	νCC (24) + νCC (15) + τHCCC (16)	
13	2919,968	6.63	νCH (39) + νCH (19)		49	866,4462	4.09	δCCC (22) + δNCN (12)	
14	2904,78	19.81	νCH (43) + νCH (51)		50	832,9659	7.67	τHCCC (13) + τHCNC (51)	863
15	1597,821	20.17	νCC (18)+ νCC (18)	1615	51	820,4071	1.20	τHCCC (17)+ τHCCC (12)+ τHCCC (21)+ τCNCN (12)+	
16	1563,035	1.77	νCC (10)		52	759,083	7.54	νCC (11) + δCNC (13) + δCNC (15)	
17	1477,889	90.25	νNC (40)	1490	53	747,8005	4.06	τHCCC (18)	
18	1463,426	5.42	δHCH (15)		54	730,0403	9.33	νNC (10) + νNC (21) + δNCN (12)	
19	1462,159	2.21	δHCH (13)		55	723,6981	78.49	τHCCC (11) + τHCCC (22)	740
20	1451,689	5.91	δHCH (19) + δHCH (28)		56	713,7788	3.15	δHCC (15) + τHCCC (21) + τHCCC (23)	
21	1451,118	8.75	δHCH (39)		57	617,3405	2.30	τCNCN (30)	
22	1442,176	3.04	δHCH (29) + δHCH (12)		58	593,8279	3.08	νCC (12) + δCNC (14)	
23	1440,667	2.15	δHCH (10) + δHCH (43) δHCH (28)		59	567,5116	0.66	τHCCC (10) + τCCCN (29)+ τCCNC (12)	
24	1430,313	46.54	δCNC (12) + δHCC (15) δHCC (17)		60	533,809	0.30	δCCC (14)	
25	1362,531	8.82	νNC (13) + δHCC (21)		61	468,9753	5.34	δCNC (26)	
26	1354,409	14.76	τHCNC (12)		62	416,5845	7.03	δCCC (16) + τCNCC (28)	
27	1344,277	25.83	νNC (12)+ νNC (24)	1300	63	415,0472	1.36	δCCC (23)	
28	1335,46	6.02	τHCNC (13)+ τHCCC (12)		64	257,0141	0.88	δCNC (21)	
29	1316,057	18.57	νCC (15)	1310	65	237,3977	0.45	τHCCC (17) + τHCCC (21)	
30	1287,014	0.34	δHCC (31)		66	218,9029	0.43	δCNC (21)	
31	1272,309	3.95	δHCC (14) + δHCC (11) + δHCC (13)		67	218,6418	1.68	τCCCN (36) + τCNCN (16)	
32	1264,603	36.54	νNC (18)	1275	68	166,7633	1.96	δCCC (22) + τCCNC (14)	
33	1251,407	5.24	τHCCC (18)		69	113,6667	0.01	τCCCC (65)	
34	1231,8	28.36	δHCC (16)	1225	70	62,98702	0.02	τCCCN (71)	
35	1184,968	30.99	δHCN (10)+ δHCC (10)	1200	71	37,4345	0.38	τCCNC (60) + τCCCC (10)	
36	1180,54	6.48	νNC (17) + δHCN (29)						

ν ;stretching. δ; bending. τ; torsion vibrations.

**Table S5**. Mulliken atomic charges of N-Butyl-1H-benzimidazole			
Atoms	Charges	Atoms	Charges

C1	0.14132	H15	0.17715
C2	-0.22164	C16	0.06192
C3	-0.32792	H17	0.1531
C4	-0.31576	H18	0.13609
C5	-0.2385	C19	-0.24316
C6	-0.20609	H20	0.12718
C7	0.305	H21	0.1295
H8	0.18349	C22	-0.61749
H9	0.15194	H23	0.13986
H10	0.17072	H24	0.14482
H11	0.13464	H25	0.13988
H12	0.18223	N26	0.20546
C13	-0.55373	N27	-0.12611
H14	0.16612		
